# Out-of-hours palliative care provided by GP co-operatives: availability, content and effect of transferred information

**DOI:** 10.1186/1472-684X-8-17

**Published:** 2009-11-28

**Authors:** Bart PM Schweitzer, Nettie Blankenstein, Luc Deliens, Henriette van der Horst

**Affiliations:** 1 Department of General Practice, and EMGO+Institute for Health and Care Research, VU University Medical Centre, Amsterdam, The Netherlands; 2 Department of Public and Occupational Health, and EMGO+Institute for Health and Care Research, VU University Medical Centre, Amsterdam, The Netherlands; 3 End-of-Life Care Research Group, Vrije Universiteit Brussels, Belgium

## Abstract

**Background:**

Out-of-hours GP care in England, Denmark and the Netherlands has been reorganised and is now provided by large scale GP co-operatives. Adequate transfer of information is necessary in order to assure continuity of care, which is of major importance in palliative care. We conducted a study to assess the availability, content and effect of information transferred to the GP co-operatives.

**Methods:**

Cross-sectional exploratory study of all palliative care phone calls during a period of one year to a GP co-operative.

**Results:**

The total number of phone calls about patients who needed palliative care was 0.75% of all calls to the GP co-operative. Information was transferred by GPs on 25.5% of palliative care patient calls, and on 12% of palliative care patient calls from residential care homes. For terminally ill patients the number of information transfers increased to 28.9%. When information was transferred, the content consisted mainly of clinical data. Information about the diagnosis and current problems was transferred in more than 90% of cases, information about the patient's wishes in 45% and information about the patient's psychosocial situation in 30.5% of cases.

A home visit was made after 53% of the palliative care calls.

When information was transferred, fewer patients were referred to a hospital.

**Conclusion:**

GPs frequently fail to transfer information about their palliative care patients to the GP co-operatives. Locums working at the GP co-operative are thus required to provide palliative care in complex situations without receiving adequate information

GPs should be encouraged and trained to make this information available to the GP co-operatives.

## Background

A great deal of palliative care, especially in the final year of a palliative care patient's life, takes place in the patient's home. In many European countries the development of palliative care has been influenced by a strong emphasis on primary care [1]. Dutch general practitioners (GPs) consider palliative care as an essential part of family medicine. In their opinion, providing care at home for dying patients is an important aspect of achieving their goal of "comprehensive, continuous and personal care" for these patients. Until 2000, most patients received out-of-hours palliative care from their own GP, and availability for home visits and out-of-hours care was identified by both patients and GPs as a core aspect of value in palliative care [2]. This availability has virtually disappeared after the recent reorganisation of out-of-hours care. In palliative care, continuity of the care is essential, and when this is no longer possible for the patient's own GP, adequate information must be transferred to locums in order to ensure good quality care [3] Palliative care is defined as the active, total care of a patient whose illness is not responsive to curative treatment. Control of pain, other symptoms, and social, psychological and spiritual problems is paramount [4].

GPs in the Netherlands have reorganised their out-of-hours care from rota groups to larger scale GP co-operatives [5]. Patients are now likely to receive out-of-hours care from a doctor, or even from several different doctors, who do not know them, and night shifts are frequently the responsibility of young doctors who work only as locums in the co-operatives.

This reorganisation in the Netherlands was preceded by reorganizations in out-of-hours primary care in the early 1990s in the United Kingdom (UK) and Denmark [6,7]. Patients in the UK, Denmark, and the Netherlands are generally positive about the care that they receive from GP co-operatives, but some people have expressed concerns regarding the care that complex, time consuming, palliative care patients receive [8,9]. A study of the experiences of patients and their carers identified the barriers in their access to care. These especially concerned patients' uncertainties and the inappropriateness of the service responses, which are mainly designed for acute medical care and do not meet the palliative care needs [10].

Recent research among Dutch GPs working as locums in a GP co-operative showed that they were unsatisfied with the quality of the information about the patient that was transferred and available to them.

(Schweitzer BPM, Blankenstein AH, Willekens M, Terpstra E, Giesen P, Deliens L. GPs' views on transfer of information about terminally ill patients to the out-of-hours co-operative. Submitted)

Although a study in the UK reported that few GPs routinely handed over information about their palliative care patients to their GP cooperatives, [11] the introduction of a dedicated fax form with which GPs can inform the co-operatives about terminally ill patients resulted in an increase of the transfer of information [12].

The complex needs of palliative care patients require a well-informed and expert response and inadequate service provision can lead to problems in symptom control and an increase in unnecessary hospital admissions. Moreover, it may leave patients and their carers confused, and inadequately supported [13].

After introduction of an out-of-hours protocol for community palliative care GPs felt that this protocol had made a positive contribution to palliative care and that the out-of-hours handover form played a key role in improving communication and the co-ordination of services [14].

In general, GPs in the UK were satisfied with the palliative care provided by their out-of-hours co-operatives, but satisfaction was less for inner-city GPs who had concerns about the continuity of care [15]. District nurses reported less satisfaction, especially with the quality of the advice, the reluctance to visit, and difficulties in obtaining medication [16].

In a survey among medical directors of GP co-operatives, only 37% believed that they could obtain specialist advice out-of-hours, although 89% of specialists said that they provided such a service. The study confirmed that in the UK there is patchy access to community nursing and palliative care services out-of-hours [18].

Patients and carers had difficulty in deciding whether or not to call out-of-hours services. Although calls were made predominantly for physical reasons, the decision to call was also strongly influenced by psychosocial factors. Positive experiences of patients were related to effective planning, in particular the transfer of information, and empathic responses from the staff [9].

The aim of this study was to investigate the transfer of information about palliative care patients to a GP co-operative and the influence of that information on the care provided by the locums in the co-operative.

The following research questions were addressed:

1. In what percentage of palliative care calls was information from the patient's own GP available in the GP co-operative?

2. Which patient characteristics are related to the transfer of information?

3. What is the content of the information transferred by the GP?

4. To what extent is the availability of patient information in the GP co-operative related to the type of contact and actions provided by the locum?

## Method

### Design: Cross-sectional exploratory study.

We performed a retrospective study of all palliative care phone calls made during a one-year period (1/11/05-1/11/06) to the GP co-operative in Amsterdam. All 424 GPs in the region of Amsterdam participate in 8 out-of-hours GP centres belonging to the Amsterdam GP co-operative. Most of the GPs work their shifts as a locum for the GP co-operative and the population served by the Amsterdam GP co-operative is 800.000 inhabitants.

We carried out an electronic search in Callmanager, which is the database of the GP co-operative, containing medical data on all calls with the GP co-operative. It also contains all information transferred by GPs about their patients on a fax form which is sent from the general practice to the co-operative and entered into the database by a medical secretary.

### Study populations

All patient related phone-calls to the Amsterdam GP co-operative between 1/11/05 and 1/11/06; all palliative care calls between 1/11/05 and 1/11/06 and the patients involved.

### Measurements

Numbers of the different types of contact following the phone-calls (telephone consultation, centre consultation, home visit) were obtained from the Annual Reports of the GP co-operative.

The records of all phone-calls in the Callmanager database during the study period were screened electronically.

We identified palliative care calls by means of a search with the text words "palliative, "terminal", "cancer", "carcinoma', "inoperable", "opioid", and "fentanyl". The 2304 identified records were subsequently examined by the researcher, and 1263 non-palliative calls were excluded. The sensitivity of the search was checked by comparing the electronic search results with hand searched data from all calls during a period of one month. This did not produce any new calls regarding palliative care patients, so we decided not to carry out a hand search for the entire study.

To answer the research questions the following data were extracted from each identified record:

Question 1: Presence or absence of information transferred by the patient's own GP.

Question 2: Patient characteristics (age, gender, residence, diagnosis as noted by the locum and terminal status (described as such by the locum).

Question 3: Content of transferred information (information about diagnosis, prognosis, medication, current problems, management plan, patient's awareness of prognosis, patient's wishes, carers and professionals involved, previous contacts, availability of own GP). A previous study showed that locums are satisfied with the quality of the transferred information if these elements are included.

Question 4: Type of contact with the locum (telephone consultation, centre consultation, home visit) and care provided by the locum (prescription or change of medication, advice only, referral for hospital admission)

The data were analyzed with SPSS 15.0. Frequencies were calculated for all variables. To determine whether patients for whom information from the GP was available and patients for whom no information was available differed from each other Chi-square tests were used for the variables gender, residence, underlying disease, terminal status, type of contact and care provided by the locum.

We used logistic regression analysis to analyze determinants for referral to hospital. The dependent variable was referral to hospital versus all other actions by the locum. The independent variables were the continuous variable age-class and the categorical variables residence (home, residential care home), terminal status (yes, no), information transfer (yes, no) and the reasons for encounter (RFE) pain, circulatory and digestive (pain, respiratory, urinary, digestive, fatigue, circulatory, psychological, other). We calculated the Exp (B) and Wald statistic for each of these parameters. The model's adequacy was determined by calculating Nagelkerke R-square.

## Results

The total number of patient calls to the GP co-operative during the one-year study period was 137.828. A total of 1041 palliative care-related calls were made to the GP co-operative during that year, concerning 553 different patients. The mean age of the patients was 74.3, the most frequently mentioned underlying disease was cancer (76.5%) and the disease was unknown in 16.1%. However, in the group of patients over 90 years of age cancer was diagnosed in 33% and the disease was unknown in 52%. According to the locums, 74.5% of all palliative care patients were terminally ill.

Information on 141 patients receiving palliative care was transferred to the GP co-operative (25.5%). (Additional file 1: Table S1) The incidence of information transfer did not differ according to the various underlying diseases, gender or age-groups, with the exception of the group of patients over 90 years of age, for 10.5% of whom information was transferred. Information was transferred for 12% of patients in residential care homes and for 28.9% of terminally ill patients.

Additional file 1: Table S2 shows the content of information transferred from GP to GP co-operative. Information on diagnosis and current problems was transferred most frequently (>90%). Information about the patient's wishes was transferred in 44.7% of cases, about carers in 41.8%, about previous contacts in 41.8%, about other professionals involved in 39% and about psychosocial aspects in 30.5%. Information about the availability of the patient's own GP (for example: mobile number of the GP) was transferred in 9.9%.

Additional file 1: Table S3 shows that in 53% of the requests for help regarding palliative care a home visit was made, while the overall percentage of home visits was 13%. It also shows that palliative care-related calls accounted for 0.75% of all calls. These calls resulted more often in a home visit than regular calls, but the presence of information did not make any difference with regard to the handling of the request by telephone or by making a home visit.

When information was transferred, patients were referred to a hospital less often. (Additional file 1: Table S4) Information had been transferred for only 8.8% of all patients referred to a hospital. Medication was prescribed by the locum for 57.2% of the palliative care patients.

Information transfer and pain as reason for encounter were factors that contributed significantly to hospital referrals. (Additional file 1: Table S5) The Nagelkerke R-square for this model was 0,209, so approximately 21% of variance was accounted for in this model.

## Discussion

### Main findings

The total number of palliative care phone calls was 0.75% of all calls to the GP co-operative. Information was transferred in 25%, and when information was transferred the content consisted mainly of clinical data. Less information was transferred about the patient's wishes and the patient's personal situation.

For patients staying in residential care homes, information transfer took place in only 12%. The majority of all palliative care calls concerned terminally ill patients, and for these patients information was relatively more often transferred.

When information was transferred fewer patients were referred to a hospital.

### Comparison with the existing literature

Although GPs are aware of the importance of information transfer, there is no evidence that they routinely alert the out-of-hours doctors to the needs of palliative care patients [15]. Previous studies have suggested that continuity of care is threatened by a lack of information in the GP co-operative [10].

In answering to a web-based questionnaire, GPs assessed the importance and quality of information transferred. They stated that information about the diagnosis, the terminally ill status of the patient, and the patient's medication was important, as was information about the treatment desired by the patient, relevant changes in the illness process, and the patient's wishes regarding end-of-life care. They also valued the transfer of information about the patient's personal situation. (Schweitzer BPM, Blankenstein AH, Willekens M, Terpstra E, Giesen P, Deliens L. GPs' views on transfer of information about terminally ill patients to the out-of-hours co-operative. Submitted)

The adoption of a dedicated fax form for GPs resulted in an increase of information transfer [12]

We found that information was transferred in only 25% of cases, and also that when information was transferred; the content mainly consisted of data on diagnosis and current problems. This reduces the quality of the information transfer.

Although the availability of out-of-hours GP care is highly valued by patients and their carers, little is known about the type of palliative care delivered by a GP co-operative [2]. In this study we found that half of the calls regarding palliative care resulted in a home visit by the locum, and that medication was prescribed in 57% of all palliative care calls.

About the relevance of information transfer: a report from the UK stated that a lack of information can lead to problems in symptom control and an increase in unnecessary hospital admissions [13] We found that when information was transferred less patients were referred to a hospital. Whether these admissions were necessary or not would be an interesting subject for further research.

Our finding that information was transferred less frequently for patients staying in residential care homes might be explained by the GP's opinion that the care for these patients and the availability of this information is the responsibility of the care home. However, few care home staff members have sufficient training in providing end-of-life care, and it is therefore important that GPs ensure the continuity of their care by providing information to the GP co-operative [18].

Less information is also transferred for the oldest patients. One reason for this maybe the complexity of conditions and co-morbidities.

Apparently it is more difficult to assess the clinical situation of these patients; in this group no diagnosis was determined for 52% of the patients.

Information was transferred more frequently when patients were terminally ill. The sense of urgency for the transfer of information is apparently greater, and these patients are more likely to be perceived as palliative care patients.

When a call is made for a palliative care patient, this patient is often already terminally ill. This suggests that the need for help, not only for physical reasons, increases in the terminal phase and waiting for care until office hours is no longer an option. It also supports the view that even more home visits should be made.

### Strengths and weaknesses of this study

In order to develop a strategy for the provision of better palliative care by GP co-operatives, we studied the current behavior of GPs with regard to the transfer of information and the consequences of that behavior. A strength of this study is that we included all calls to the GP co-operative regarding palliative care during a period of one year. We studied the availability of information about all patients for whom a call was made. However, a limitation is that we do not know how many times information was transferred for patients for whom no call was made.

From the results of this cross-sectional study we can not determine whether there is a causal relationship between less hospital referrals and the transfer of information.

## Conclusion

Despite the importance of continuity of care in the terminal phase, GPs do not transfer information for the majority of their palliative care patients. If information is transferred to the GP co-operatives, the content is mainly limited to clinical data. Information about the patient's personal situation and wishes is often lacking.

Locums working in the GP co-operative are thus required to provide palliative care in complex situations without receiving adequate information. They might be better supported if this information is made available and (perhaps unnecessary) hospital admissions could possibly be avoided.

## Recommendations

GP co-operatives need to develop and implement an effective system of patient information management. GPs need to be made aware of the disadvantages of not transferring information about their palliative care patients to the GP co-operative, and should be trained to do this an adequate way. If an electronic patient file is accessible during the out-of-hours period, this should contain a specific transfer section containing information that is relevant for locums. Hence, there are potentials for improvement in the end-of-life care that is provided by the GP co-operatives.

## Competing interests

The authors declare that they have no competing interests.

## Authors' contributions

BS carried out the search and drafted the manuscript. NB participated in the analysis and draft of the manuscript. LD and HH participated in the design of the study and helped to draft the manuscript. All authors read and approved the final manuscript.

## Pre-publication history

The pre-publication history for this paper can be accessed here:

http://www.biomedcentral.com/1472-684X/8/17/prepub

## Supplementary Material

Additional file 1 **Supplemental tables**. This DOC file contains Table S1, S2, S3, S4, S5. Click here for file
