# Supplementary material for: Out-of-hours palliative care provided by GP co-operatives: availability, content and effect of transferred information
Source: BMC Palliat Care. 2009 Nov 28;8:17. doi: 10.1186/1472-684X-8-17 (PMC2789043; doi:10.1186/1472-684X-8-17)
Supplement: Additional file 1 — Supplemental tables. This DOC file contains Table S1, S2, S3, S4, S5. [file 1472-684X-8-17-S1.doc]

Table 1. Information tranfer to the GP co-operative and patient characteristics

| **Patient characteristics** | **No**  **information transferred**  **N= 412 (74.5%)** | **Information**  **transferred**  **N =141 (25.5%)** | **P**  **value** |
| --- | --- | --- | --- |
| **Sex** |  |  | 0.459 |
| Male | 216 (73.2%) | 79 (26.7%) |  |
| Female | 196 (76.0%) | 62 (24.0%) |  |
| **Age (years)** |  |  | 0.220 |
| <60 | 79 (72.5%) | 30 (27.5%) |  |
| 60-70 | 78 (69.7%) | 34 (30.3%) |  |
| 71-80 | 108 (72.8%) | 29 (27.2%) |  |
| 81-90 | 96 (69.6%) | 42 (30.4%) |  |
| >90 | 51 (89.5%) | 6 (10.5%) |  |
| Mean (SD) |  |  |  |
| **Residence** |  |  | 0.002 |
| Home | 339 (72.1%) | 131 (27.9%) |  |
| Care home | 73 (87.9%) | 10 **(**12.1%) |  |
| **Underlying disease** |  |  | 0.951 |
| Cancer | 298 (70.4%) | 125 (29.6%) |  |
| Heart failure | 16 (76.1%) | 5 (23.9%) |  |
| COPD | 11 (73.3%) | 4 (26.7%) |  |
| Neurological | 3 (60.0%) | 2 (40.0%) |  |
| Unknown | 84 (94.3%) | 5 ( 5.7%) |  |
| **Terminal status** |  |  | 0.003 |
| Terminally ill | 282 (71.0%) | 115 (29.0%) |  |
| Not terminally ill | 130 (83.3%) | 26 (16.7%) |  |

# Table 2. Content of information transferred by patient’s GP to GP co- operative

| **Content (N=141)** | **%*** |
| --- | --- |
|  |  |
| **Diagnosis** | **97.2** |
| **Current problems** | **90.1** |
| **Medication** | **84.4** |
| **Management plan** | **72.3** |
| **Prognosis** | **52.5** |
| Patient’s wishes | **44.7** |
| **Information about carers** | **41.8** |
| **Previous contacts** | **41.8** |
| **Patient’s awareness of prognosis** | **40.4** |
| **Other professionals involved** | **39.0** |
| **Psychosocial aspects** | **30.5** |
| **Availability own GP** | **9.9** |

*** % of information transfer forms in which this item was represented**

# Table 3. Palliative care related calls and information transfer to the GP co-operative

**Total number of patient calls: N=137828**

Telephone consultation 71672 ( 52%)

Centre consultation 48239 ( 35%)

Home visit 17917 ( 13%)

**Consultation**

**Centre**

**Consultation**

**Home visit**

**Palliative care calls: N=1041 (0.75%)**

Telephone consultation 481 ( 46%)

Centre consultation 9 ( 0.9%)

Home visit 551 ( 53%)

**Patients involved in palliative care calls : N=553**

Telephone consultation 255 ( 46%)

Centre consultation 5 (0.9%)

Home visit 293 ( 53%)

Patients with transferred information : N =141

Telephone consultation 69 ( 49%)

Centre consultation 0 ( 0%)

Home visit 72 ( 51%)

Table 4. Information tranfer to the GP co-operative and action by the locum

|  | **No information transferred**  **N = 412 ((74,5%)** | **Information**  **Transferred**  **N =141 (25,5%)** | **P**  **value** |
| --- | --- | --- | --- |
| **Action by locum** |  |  | 0.009 |
| Advice without medication | 120 (73.1%) | 44 (26.9%) |  |
| Medication prescribed | 227 (71.8%) | 89 (28.2%) |  |
| Referral to hospital | 62 (91.2%) | 6 ( 8.8%) |  |

**Table 5. Logistic regression: determinants of referral to hospital**

|  | OR | 95%CI | Wald | p-value | OR  unadjusted | 95%CI  unadjusted |
| --- | --- | --- | --- | --- | --- | --- |
| Residence (home vs carehome)  Information transfer (yes vs no)  Terminal status (yes vs no)  Age class (younger vs older)  Cancer (yes vs no)  Pain as RFE (yes vs no)  Circulatory problems as RFE (yes vs no)  Digestive problems as RFE (yes vs no) | 1,984  0,258  0,621  0,798  1,290  0,480  6,984  1,627 | 0,514-7,659  0,106-0,628  0,355-1,087  0,627-1,016  0,353-4,721  0,235-0,978  1,168-41,774  0,804-3,294 | 0,988  8,925  2,783  3,343  0.148  4,087  4,536  1,829 | 0,320  0.003  0,095  0.067  0,700  0.043  0.033  0,176 | 4,204  0,252  0,420  0,683  1,983  0,377  14,937  2,646 | 1,289-13.716  0107-0,598  0,250-0,706  0,553-0,842  0,590-6,660  0,197-0,772  2,682-83,195  1,406-4,980 |
| Nagelkerke R^2^ 0.209 |  |  |  |  |  |  |
